# Deceased donor kidney transplantation in candidates with pre-transplant hematological malignancies: a literature review and recipient allocation proposal in Singapore

**DOI:** 10.1007/s40620-025-02381-8

**Published:** 2025-08-22

**Authors:** Emmett Tsz Yeung Wong, Ian Tatt Liew, Hein Than, Aloysius Yew Leng Ho, Chandramouli Nagarajan, Yeow Tee Goh, Charles Thuan Heng Chuah, Michelle Limei Poon, Wee Joo Chng, Melissa Gaik Ming Ooi, Widanalage Sanjay Prasad De Mel, Allen Eng Juh Yeo, Terence Kee, Anantharaman Vathsala

**Affiliations:** 1 Department of Medicine, Yong Loo Lin School of Medicine, National University of Singapore, Level 8, NUHS Tower Block, 1E Kent Ridge Road, Singapore, 119228 Singapore; 2 National University Centre for Organ Transplantation, National University Hospital, Singapore, Singapore; 3 Department of Renal Medicine, Singapore General Hospital, Singapore, Singapore; 4 Singhealth Duke-NUS Transplant Centre, Singapore Health Services, Singapore, Singapore; 5 Department of Haematology, Singapore General Hospital, Singapore, Singapore; 6 Singhealth Duke-NUS Blood Cancer Centre, Singapore Health Services, Singapore, Singapore; 7 National Cancer Centre Singapore, Singapore, Singapore; 8 National University Cancer Institute, Singapore, Singapore, Singapore; 9 Cancer Science Institute of Singapore, Singapore, Singapore; 10 Department of Paediatrics, Yong Loo Lin School of Medicine, National University of Singapore, Singapore, Singapore; 11 Department of Paediatrics, Khoo Teck Puat - National University Children’s Medical Institute, National University Hospital, Singapore, Singapore

**Keywords:** Deceased donor kidney transplantation, Recipient selection, Cancer/malignancy, Waitlist candidacy, Consensus opinion, Patient safety

## Abstract

Deceased donor kidneys are a scarce national resource, and principles of utilitarianism and justice govern allocation. Kidney transplant recipients with a prior history of cancer show an increased risk of malignancy- and non-malignancy-related mortality compared to their counterparts without a previous history of malignancy. The inferior survival of a recipient with pre-transplant malignancy questions the allocation of a scarce resource to a population at anticipated poorer patient and graft survival. However, patient survival has significantly improved with advances in therapeutics for hematological malignancies, which led to an updated consensus expert opinion by the American Society of Transplantation in 2019. Nevertheless, the candidacy of patients with pre-transplant hematological malignancies in countries with a scarcity of deceased donor kidneys and a prolonged wait time may warrant specific considerations. This review details the basis for evaluation and candidacy recommendations for patients with a history of hematological malignancy for waitlist placement for deceased donor kidney transplantation, while optimizing scarce deceased donor organ supply in  Singapore. It considers the available evidence in countries where organ scarcity is a distinct challenge; thus, this consensus report is tailored to these constraints and may not be fully generalizable to other countries or transplant allocation algorithms.

**Graphical Abstract:**

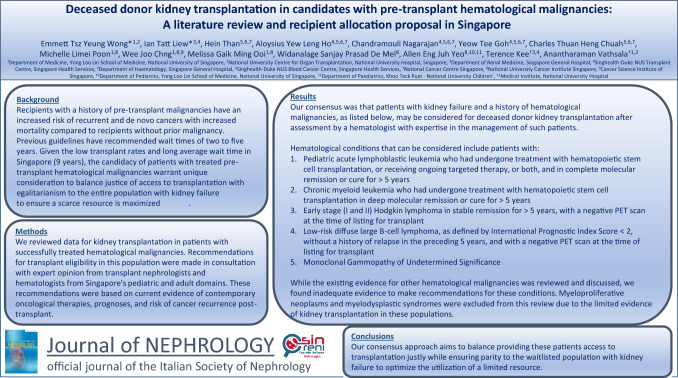

**Supplementary Information:**

The online version contains supplementary material available at 10.1007/s40620-025-02381-8.

## Introduction

Immunosuppressive therapy for kidney transplantation markedly increases the likelihood of developing malignancies in transplant recipients compared to waitlisted patients [1]. Recipients with a history of pre-transplant malignancies have an increased risk of recurrent and de novo cancers with increased malignancy- and non-malignancy-related mortality compared to recipients without prior malignancy [2]. Previous guidelines have recommended wait times of two to five years, depending on the type of pre-transplant malignancy [3].

Singapore's incident rate of treated kidney failure is amongst the highest globally, with 366 per million population (pmp) [4]. Contrariwise, rates of deceased donor kidney transplants are only 6.59 pmp, largely attributed to rigorous gun control laws, low rates of substance abuse and low incidence of accidental deaths [5]. Consequently, the average wait time for a transplant in Singapore is 9 years, exceeding that in the United States (5 years), United Kingdom (2–3 years) and Australia (2.2 years) [6–9]. Given the low transplant rates, patients with a likelihood of worse survival due to co-morbidities are usually excluded from transplantation, and deceased donor kidneys are preferentially allocated to those without significant cerebrovascular and cardiovascular diseases. Notably, allocation of deceased donor kidneys to patients with prior malignancy is restricted to those whose malignancy has presumably been cured, with similar predicted post-transplant survival to those without malignancy. Thus, the allocation of a deceased donor kidney to a recipient with pre-transplant malignancy with potentially inferior survival raises ethical questions on allocation of a scarce resource to a population at anticipated poorer patient-allograft survival. As such, candidates with pre-transplant hematological malignancies were hitherto excluded from the waitlist in Singapore regardless of their time in remission.

Over the last two decades, advances in therapeutics for hematological malignancies have significantly improved patient survival and outcomes. Patients are more likely to survive the malignancy but may present with subsequent kidney failure from other or related causes with the need for kidney replacement therapy. The anticipated expansion of cancer survivors with secondary kidney failure cannot be neglected as the overall survival of this population improves. Waitlisting candidates with prior hematological malignancies for deceased donor kidney transplantation is ethically challenging as there is a need to balance justice of access to transplantation with egalitarianism to the entire kidney failure population to ensure that benefit from scarce resource is maximized. For solid organ malignancy, a 5-year cancer survival rate of 80% was used by the American Society of Transplantation (AST) 2021 Consensus as an acceptable benchmark before proceeding with transplantation [10]. A consensus expert opinion on the timing of solid organ transplantation in patients with pre-transplant hematological malignancies was published in 2021 following The Malignancy and Transplantation Meeting held by the AST in 2019 [11]. However, the candidacy of these patients in countries with a long wait time for a deceased donor kidney may warrant specific considerations. This review details the basis for evaluation and candidacy recommendations for these patients for waitlist placement for deceased donor kidney transplantation in Singapore. While the consensus report is tailored to the context of Singapore’s deceased donation constraints and may not be fully generalizable to other countries or be transposed into other transplant allocation algorithms, it may be considered for application in contexts experiencing similar long waiting times due to low deceased donor kidney transplant rates.

## Methods

A comprehensive search was conducted for randomized controlled trials, case–control studies, case series, and cohort studies evaluating hematological malignancies in kidney transplant recipients. The search was restricted to studies published in English from January 1, 1990, to July 31, 2024, across the following databases: PubMed, Cochrane Central Register of Controlled Trials, and ClinicalTrials.gov. Additionally, epidemiological data extracted from registry databases and previously published guidelines on hematological malignancies, both within and outside the context of solid organ transplantation, were reviewed. References from retrieved articles were manually screened to identify additional relevant publications.

Recommendations for transplant eligibility were formulated based on available data and expert consensus from transplant nephrologists and hematologists specializing in both pediatric and adult care in Singapore. These recommendations were guided by current evidence on contemporary oncological therapies, disease prognoses, and the risk of cancer recurrence following transplantation.

In this review, we detail our recommendations for the inclusion of kidney failure patients with a history of treated pediatric acute lymphoblastic leukemia, chronic myeloid leukemia, lymphoma (Hodgkin and non-Hodgkin), low-risk diffuse large B-cell lymphoma, and monoclonal gammopathy of undetermined significance. We also reviewed and discussed the existing evidence for other hematological malignancies, including chronic lymphocytic leukemia, acute myeloid leukemia, monoclonal gammopathy of undetermined and renal significance, multiple myeloma, and amyloid light chain amyloidosis, but found inadequate evidence to make recommendations for these conditions. Myeloproliferative neoplasms and myelodysplastic syndromes were excluded from this review due to the limited evidence of kidney transplantation in these populations.

## Results

### Leukemias

#### Acute lymphoblastic leukemia

Acute lymphoblastic leukemia is a malignant proliferation of lymphoid progenitor cells in the bone marrow, blood, and extramedullary sites. It is diagnosed by the presence of ≥ 20% lymphoid/undifferentiated blasts in the bone marrow. The age at onset of acute lymphoblastic leukemia follows a bimodal distribution, with the first peak occurring in childhood at five (60–80% of acute lymphoblastic leukemia cases) and the second peak around 50 years of age [12, 13].

Treatment involves four phases: induction, consolidation, intensification, and maintenance. It usually comprises a combination of agents, including glucocorticoids, vincristine, tyrosine kinase inhibitors, and intrathecal chemotherapy. Hematopoietic stem cell transplantation (HSCT) is now mainly reserved for patients with high-risk disease, relapse, or persistent minimal residual disease [14]. Long-term treatment effects include endocrinological, cardiovascular, and cognitive disorders, infections, and secondary malignancies, which significantly impact survivor mortality.

The 5 year overall survival for acute lymphoblastic leukemia varies between children and adolescents (89%), young adults (61%) and adults (< 45%) [15–17]. Relapse occurs in 15–20% of patients within 2.5 years after treatment in most cases, and portends a guarded prognosis (median overall survival: 4.5 months, 5 year overall survival: 10% following a relapse) [18].

Recipients who underwent kidney transplantation following HSCT reported favorable outcomes with 100% allograft and patient survival (Supplementary Table 1) [19–21]. To date, there have been no reports of tyrosine kinase inhibitor-treated acute lymphoblastic leukemia followed by kidney transplantation. Advances in chemotherapy have shifted HSCT to a salvage therapy for high-risk patients. Limiting transplantation to only those who have undergone HSCT may inadvertently select patients with higher recurrence risk and potentially poorer outcomes.

Based on the precedence of prior successful kidney transplants and the favorable long-term prognosis of treated pediatric acute lymphoblastic leukemia, our consensus was that patients with kidney failure and a history of successfully treated pediatric acute lymphoblastic leukemia, whether through HSCT or targeted therapy (including those currently undergoing treatment with tyrosine kinase inhibitors), eligible for waitlist placement after assessment by a hematologist and determined to have achieved complete molecular remission for at least five years with documented stability.

#### Chronic lymphocytic leukemia

Chronic lymphocytic leukemia is a B-cell lymphoproliferative disorder with a variable progression. Presentation varies from asymptomatic indolent disease to rapid, symptomatic disease progression, with or without bone marrow failure. Chronic lymphocytic leukemia may result in kidney injury due to direct lymphomatous infiltration of the kidney, tumor lysis, paraproteins (immunotactoid, fibrillary, or cryoglobulin), thrombotic microangiopathy, and granulomatous interstitial nephritis [22]. Chronic lymphocytic leukemia is characterized by relapses, even after prolonged response to therapy [23].

A scoping review found 13 kidney transplant recipients with prior chronic lymphocytic leukemia, which progressed or recurred in five (39%) cases. Allograft loss was reported in two (15%) cases at 14- and 61-months post-transplant. Six (46%) deaths were reported, with patients having recurrent infectious complications [24–27].

While the AST 2021 guidelines recommend an interval of 2–3 years after completing treatment of chronic lymphocytic leukemia [11], given the unfavorable outcomes of kidney transplantation in these patients, the consensus was to maintain the exclusion of patients with a prior history of chronic lymphocytic leukemia from the waitlist.

#### Acute myeloid leukemia

Acute myeloid leukemia is characterized by a clonal proliferation of primitive hematopoietic stem cells or progenitor cells of the myeloid lineage. This results in a neoplastic population of myeloid blasts infiltrating the bone marrow, with varying degrees of differentiation based on the subtype of acute myeloid leukemia. The disease predominantly occurs in older persons (> 60 years of age). It is diagnosed by the presence of ≥ 20% blasts in the peripheral blood or bone marrow or the presence of acute myeloid leukemia-defining genetic abnormalities regardless of blast count [for example, t(8;21), inv(16), or t(15;17)].

Induction chemotherapy (daunorubicin, cytarabine) is utilized in young fit patients by adding targeted agents such as midostaurin and gemtuzumab in specific genomic subtypes. Consolidation therapy includes high-dose cytarabine in favorable-risk patients or HSCT in high-risk patients. Patients who are not fit for intensive induction therapy may receive treatment with azacitidine and venetoclax. Prognosis varies based on the genomic subtype, with cure rates of over 60% for patients with a favorable genetic profile, while high-risk patients have a poor outcome.

To date, there have been no reports of acute myeloid leukemia treated with chemotherapy or targeted therapy followed by kidney transplantation. Given the vintage of HSCT as a treatment option, there have been seven case reports in a scoping review of patients with acute myeloid leukemia treated with HSCT followed by kidney transplantation [19, 28]. One death was reported from secondary metastatic squamous cell carcinoma of the vagina. None of the recipients experienced allograft loss.

Given the overall poorer prognosis of acute myeloid leukemia compared to acute lymphoblastic leukemia and the absence of contemporary data, our consensus was to continue to exclude patients with acute myeloid leukemia from the kidney transplant waitlist in the Singapore context until sufficient data become available for review. Where organ donor scarcity is less of a consideration, deceased donor kidney may be considered for candidates deemed to have been cured for more than five years.

#### Chronic myeloid leukemia

Chronic myeloid leukemia is an abnormal myeloid proliferation in the bone marrow secondary to a translocation between chromosomes 9 and 22, t(9;22)(q34; q11.2) forming the BCR-ABL fusion gene. BCR-ABL encodes a constitutively active fusion BCR-ABL oncoprotein, resulting in continuous cell proliferation, apoptosis inhibition, and cellular adhesion deregulation. The median age at onset of chronic myeloid leukemia ranges from 45–56 years, with a male predominance [29, 30].

First-line therapy involves a first-generation tyrosine kinase inhibitor (imatinib) or second-generation tyrosine kinase inhibitors (nilotinib, dasatinib, or bosutinib). In patients with resistance or intolerance to first-line treatment, second-generation tyrosine kinase inhibitors (if a first-generation tyrosine kinase inhibitor had been used) or a third-generation tyrosine kinase inhibitor (ponatinib) may be used. Allogeneic HSCT is recommended as salvage therapy for selected patients if resistance has developed after at least two lines of tyrosine kinase inhibitor treatment or if the disease progressed from the chronic phase to a more advanced one [31]. Chronic myeloid leukemia is highly treatment-responsive, with a 10-year survival with imatinib ranging from 72–98% [29, 31].

Kidney transplantation has been successfully performed in patients whose chronic myeloid leukemia was treated with HSCT or tyrosine kinase inhibitors. These recipients have favorable outcomes with 100% allograft and patient survival, with no chronic myeloid leukemia recurrence post-transplantation (Supplementary Table 2) [19, 20, 32–34].

Based on the precedence of prior successful kidney transplantation and the favorable long-term prognosis of treated chronic myeloid leukemia, the consensus was that patients with kidney failure and a history of successfully treated chronic myeloid leukemia with HSCT may be considered for waitlist placement. This eligibility is contingent on an assessment by a hematologist and the achievement of sustained deep molecular remission corresponding to a BCR-ABL transcript level of less than 0.01% on the International Scale (i.e., MR^4^ or better) [31] for at least five years with documented stability. Given the limited data on outcomes after kidney transplantation in patients with chronic myeloid leukemia receiving ongoing tyrosine kinase inhibitor therapy, our consensus was to defer their inclusion until more evidence becomes available.

### Lymphomas

Lymphomas are a heterogeneous group of malignancies that arise from the clonal proliferation of B-cell, T-cell, or NK-cell subsets of lymphocytes at different stages of maturation. Lymphomas are classified into non-Hodgkin's lymphoma (90%) and Hodgkin's lymphoma (10%). Globally, they were the 10th and 26th most frequent incident cancers and the 11th and 28th most frequent cause of cancer deaths in 2022, respectively [35].

#### Hodgkin lymphoma

Hodgkin lymphoma is a lymphoid neoplasm with pathognomonic multinucleated Reed–Sternberg cells admixed with a population of non-neoplastic inflammatory cells with or without fibrosis. Initial treatment for Hodgkin lymphoma is selected based on the presenting stage (Lugano classification, a modification of the Ann Arbor staging system) and prognostic factors [36]. Age-standardized 1 year, 5 year, and 10 year overall survival for adults with Hodgkin lymphoma are 90–92%, 82–87%, and 75–80%, respectively, in the UK and the US [37, 38].

Patients with early-stage Hodgkin lymphoma (Lugano stages I and II) are stratified into favorable and unfavorable prognoses based on prognostic features such as age, B symptoms, extent of disease, and mediastinal adenopathy. The intensity of chemotherapy (commonly ABVD—doxorubicin, bleomycin, vinblastine, and dacarbazine, with or without Brentuximab Vedotin) and involved-site radiation therapy is titrated according to the stratification of the disease. These patients are highly likely to achieve long-term complete remission. The five-year relative survival rate was 93% for localized Hodgkin lymphoma in patients diagnosed in the US from 2012 to 2018 [39].

Advanced-stage Hodgkin lymphoma refers to stage III and IV disease, although many experts and clinical trials have included patients with stage II with bulky nodal disease. Combination chemotherapy is the mainstay of treatment, while radiation therapy may be used for selected patients as consolidation. The 5-year survival rates for advanced-stage Hodgkin lymphoma are 65–80%. Prognosis is primarily determined by the International Prognostic Score (IPS). Primary refractory Hodgkin lymphoma and relapsed Hodgkin lymphoma were hence excluded from our review for suitability for waitlist placement due to their poorer prognosis.

Most patients with Hodgkin lymphoma attain complete remission after initial treatment and achieve long-term disease control. Relapses occur in 10–15% of patients with favorable early-stage Hodgkin lymphoma and 15–30% of patients with advanced-stage Hodgkin lymphoma. Non-ABVD chemotherapy regimens, advanced-stage disease at the time of initial presentation, high IPS, and bulky disease are risk factors associated with relapsing disease [40]. More than half of recurrences occur within two years of the primary treatment, and up to 90% occur before the 5 year mark [41].

#### Non-Hodgkin lymphoma

Non-Hodgkin lymphomas are a diverse group of hematologic malignancies derived from mature B-cells, T-cells, or NK cells. Treatment depends on the subtype. Non-Hodgkin lymphoma prognosis is primarily determined by histopathology, with clinical factors such as age, extra-nodal disease, performance status, and disease stage playing a secondary role [42].

Many patients with diffuse large B-cell lymphoma achieve a long-term disease-free status with aggressive combination chemotherapy. The standard treatment for diffuse large B-cell lymphoma is R-CHOP (Rituximab, cyclophosphamide, doxorubicin, vincristine, and prednisone) or Polatuzumab R-CHP [43, 44]. Reimaging by positron emission tomography (PET)/computed tomography (CT) is recommended to assess treatment response at the end of the treatment. Most relapses occur within two years of treatment [45]. Diffuse large B-cell lymphoma is cured with current therapy in approximately half of cases, particularly in those who achieve complete remission with first-line treatment. Prognosis is stratified by the International Prognostic Index (IPI), with a conventional IPI of ≤ 2 considered low-intermediate risk with a 4-year progression-free survival and overall survival of 80% and 81%, respectively [46].

Our expert panel considered various other subtypes of non-Hodgkin lymphoma, including follicular, mantle cell, Burkitt, and peripheral T-cell lymphomas. Collectively, these lymphomas were associated with a less favorable prognosis than diffuse large B-cell lymphoma—they had a more aggressive clinical course, poorer survival, and/or higher risk of relapse [47–49]. Therefore, patients with a history of non-Hodgkin lymphoma of subtypes other than low-risk diffuse large B-cell lymphoma were excluded from consideration for the waitlist.

There is limited literature and experience of solid organ transplantation in Hodgkin lymphoma and non-Hodgkin lymphoma. One of the largest series examining patients with a history of lymphoma who underwent kidney transplantation is the Israel Penn International Transplant Tumour Registry. In this retrospective review of 91 patients with a history of lymphoma who underwent solid organ transplantation (34% kidney) from 1968 to 2001, the median disease-free interval pre-transplant was 99 months [50]. Eight (out of 81, 10%) had a recurrence (3 Hodgkin lymphoma and 5 out of 47 [11%] non-Hodgkin lymphoma) during a median follow-up of 25.7 months post-transplant. Recipients with less than two years between diagnosis and transplantation had an increased risk of relapse, with 75% of relapses occurring within two years of transplantation. The 5 year overall survival after transplantation was 68% in recipients with a history of Hodgkin lymphoma and 85% for non-Hodgkin lymphoma. Survival after recurrence was poor (one in three [33%] Hodgkin lymphoma and one in five [20%] non-Hodgkin lymphoma), with a median survival of 6.8 months.

A recent review recognized that most authorities recommend a 2- to 5-year waiting period before a patient with treated lymphoma undergoes transplantation based on the observation that most recurrences occurred within this period [51]. It also recommended that patients with a history of lymphoma be evaluated with fluorodeoxyglucose (FDG)–PET imaging before placement on a transplant waitlist to exclude occult relapse.

While the AST 2021 guidelines recommend a uniform interval of 2 years to transplant after achieving progression-free survival for diffuse large B-cell lymphoma or event-free survival for Hodgkin lymphoma, follicular, Burkitt, or other peripheral T lymphomas, our group adopted a differential approach to these lymphomas as the prognosis for each was different [11]. Thus, we considered waitlist placement for kidney failure patients with prior Hodgkin lymphoma and diffuse large B-cell lymphoma. Our consensus was that kidney failure patients with a history of prior Hodgkin lymphoma may be considered for waitlist placement after assessment by a hematologist with expertise in the management of such patients if the patient 1) presented with early-stage Hodgkin lymphoma (stages I and II); 2) has been in stable remission for at least five years; and 3) has a negative PET scan at the time of waitlist placement. Where organ donor scarcity is less of a consideration, candidates with advanced Hodgkin lymphoma and responding to chemotherapy with remission for more than five years, may be considered as deceased kidney donors.

Kidney failure patients with a history of prior low-risk diffuse large B-cell lymphoma, as defined by IPI ≤ 2, may be considered for waitlist placement after assessment by a hematologist with expertise in the management of such patients, and if the patient 1) had no history of relapse in the preceding five years; and 2) has a negative PET scan at the time of waitlist placement.

### Plasma-cell dyscrasias

Monoclonal gammopathies, multiple myeloma, and light chain amyloidosis are plasma cell dyscrasias characterized by clonal proliferation of plasma cells, monoclonal immunoglobulin secretion, and often kidney impairment. Over the last two decades, the development and implementation of novel therapies such as proteasome inhibitors and CD38-directed monoclonal antibodies have improved the hematologic outcomes of both initial and relapsed disease. When combined with autologous HSCT, the outcomes of kidney transplantation in plasma cell dyscrasia patients have improved [52].

#### Monoclonal gammopathy of undetermined significance

Monoclonal gammopathy of undetermined significance has an estimated prevalence of 1.7–6.5% [53]. It is a clinically asymptomatic premalignant clonal plasma cell or lymphoplasmacytic proliferative disorder defined by immunoglobulin (M protein) concentration less than 3 g/dl, plasma cells in the bone marrow less than 10%, and absence of myeloma-defining features or symptoms.

No treatment is required for monoclonal gammopathy of undetermined significance, which has a malignant transformation rate of 1% per year [53]. Multiple studies have attempted to define the risk of monoclonal gammopathy of undetermined significance progression to a malignant entity after transplantation. In a Mayo Clinic series, 42 cases of monoclonal gammopathy of undetermined significance were identified among 3518 patients who underwent kidney transplantation over > 40 years [54]. Twenty-three (55%) were diagnosed pre-transplant, and 19 (45%) were diagnosed post-transplant. During a median follow-up of 8.5 years, four (17%) recipients with pre-transplant monoclonal gammopathy of undetermined significance progressed to hematologic malignancy — two developed post-transplant lymphoproliferative disorder and two smoldering myelomas. Together with other cohort studies, they indicate a low rate of progression of monoclonal gammopathy of undetermined significance to multiple myeloma and lymphoproliferative disorders after transplant and provide evidence for the relative safety of kidney transplantation among patients with monoclonal gammopathy of undetermined significance [54, 55].

As such, our consensus was that kidney failure patients with a history of monoclonal gammopathy of undetermined significance may be considered for waitlist placement after assessment by a hematologist with expertise in the management of such patients. These waitlisted patients and subsequent transplant recipients should be monitored by a hematologist with expertise in the management of such patients, at least annually, to monitor for the progression of monoclonal gammopathy of undetermined significance.

#### Monoclonal gammopathy of renal significance

Monoclonal gammopathy of renal significance-associated kidney diseases encompass a broad spectrum of kidney pathology, including monoclonal immunoglobulin deposition diseases, light chain deposition disease, heavy chain deposition disease, C3 glomerulopathy with monoclonal gammopathy, and several others. The incidence and prevalence of the various types of monoclonal gammopathy of renal significance are generally unknown.

The treatment of monoclonal gammopathy of renal significance is determined primarily by the pathologic type of kidney injury, the nature of the clone producing the nephrotoxic monoclonal immunoglobulin, and the likelihood of reversing existing kidney damage or preventing further kidney injury. Although evidence is limited, several studies of patients with monoclonal gammopathy of renal significance have shown that kidney outcomes are closely associated with the hematologic response to chemotherapy targeted at plasma cells or other B-cell neoplasms [56–58].

A case series at the Mayo Clinic comprising patients with light chain deposition disease, C3 glomerulopathy with monoclonal gammopathy, and light chain proximal tubulopathy showed that recurrence is common in all monoclonal gammopathy of renal significance-associated lesions after kidney transplantation [59]. A complete hematologic response may reduce the risks of recurrence, allograft loss, and death.

#### Multiple myeloma

Multiple myeloma has an estimated global incidence of 5.4–53 pmp [60]. Myeloma-dependent cast nephropathy is a leading cause of acute kidney injury in patients with multiple myeloma and is defined as a myeloma-defining event.

Standard therapy for multiple myeloma involves novel agent-based induction therapy followed by autologous HSCT and maintenance therapy for transplant-eligible patients [61]. Transplant-ineligible patients are treated with induction followed by maintenance therapy. Current induction regimens may include proteasome inhibitors, immunomodulators, and monoclonal antibodies [62].

Experience of kidney transplantation following myeloma remission is limited to case reports. In a case series of 12 kidney transplant recipients after treatment of plasma cell dyscrasias with HSCT (2 monoclonal gammopathy of renal significance, 10 multiple myeloma), all patients had a functioning allograft after one year (median follow-up of 44 months) [63]. Nine of the 12 patients were alive and had a functioning allograft five years after transplant. Five patients experienced a relapse of plasma cell dyscrasias (of whom three responded well to subsequent therapies), and four developed secondary malignancies. In another case series of 12 kidney transplant recipients with treated multiple myeloma, with a median follow-up of 40 months after transplant, hematologic progression occurred in nine (75%) transplants [64]. Three (25%) allografts failed, and five (45.5%) recipients experienced death with functioning allografts. Allograft survival at 1 and 5 years was 82.5% and 66%, respectively. Overall survival rates of the cohort at 1, 3, and 5 years were 81.8%, 61.4%, and 61.4%, respectively. All deaths occurred owing to hematologic progression or treatment-related complications.

Overall, outcomes of kidney transplantation in multiple myeloma were mixed, demonstrating that although kidney disease attributed to plasma cell dyscrasias may have favorable outcomes after transplant, relapse remains common [52, 55, 65, 66].

#### Amyloid light chain amyloidosis

Light chain amyloidosis has an estimated global incidence of 10 pmp [67]. It is a systemic disease characterized by the extracellular deposition of congophilic fibrils in soft tissues. Patients with low tumor burden should be considered for upfront autologous HSCT. In contrast, those ineligible for upfront HSCT due to high tumor burden may first receive systemic therapy with subsequent reassessment for HSCT based on functional status with or without organ response [68]. Both strategies employ systemic therapy to eliminate the plasma cells responsible for synthesizing immunoglobulin light chains.

The limited available experience for kidney transplantation in light chain amyloidosis demonstrates significant rates of recurrence in the allograft or extrarenal organs. Studies published in 2010 by a group from the UK and in 2011 by the Mayo Clinic showed that recurrence of amyloidosis occurred in 5/22 and 2/19 transplant recipients, even though the allograft was still functioning at a median of 4.8 years and 41.4 months, respectively [69, 70].

While the AST 2021 guidelines suggested that some kidney failure patients with multiple myeloma or light chain amyloidosis meeting stringent criteria could undergo kidney transplantation [11], our consensus was to exclude patients with these conditions from consideration for the kidney transplant waitlist due to the significant risk of recurrence of monoclonal gammopathy of renal significance, multiple myeloma, and light chain amyloidosis.

After reviewing the currently available data, our consensus was that patients with kidney failure and a history of treated pediatric acute lymphoblastic leukemia, chronic myeloid leukemia, Hodgkin lymphoma, low-risk diffuse large B-cell lymphoma, and monoclonal gammopathy of undetermined significance could be considered for transplant waitlist placement after assessment by a hematologist with expertise in the management of such patients (Table 1). Where organ donor scarcity is less of a consideration, candidates with acute myeloid leukemia who have favorable cytogenetics and those with advanced Hodgkin lymphoma, who are deemed durably cured for more than five years, may also be considered as deceased kidney donors.

**Table 1:** Summary of recommendations

Summary of recommendations
Patients with kidney failure and a history of hematological malignancies, as listed below, may be considered for deceased donor kidney transplantation after assessment by a hematologist with expertise in the management of such patients Hematological conditions that can be considered include:
Pediatric acute lymphoblastic leukemia (ALL) patients who had undergone treatment with hematopoietic stem cell transplantation (HSCT), or receiving ongoing targeted therapy, or both, and in complete molecular remission or cure for > 5 years
Chronic myeloid leukemia (CML) patients who had undergone treatment with HSCT in deep molecular remission or cure for > 5 years
Early stage (I and II) Hodgkin lymphoma patients in stable remission for > 5 years, with a negative PET scan at the time of listing for transplant
Low-risk diffuse large B-cell lymphoma (DLBCL) patients, as defined by International Prognostic Index Score < 2, without a history of relapse in the preceding 5 years, and with a negative PET scan at the time of listing for transplant
Monoclonal Gammopathy of Undetermined Significance (MGUS)
Kidney failure patients waitlisted for transplant and transplanted recipients should be monitored by a hematologist with expertise in the management of such patients, at least annually, to monitor for recurrence Kidney transplant recipients with a pre-transplant malignancy should have appropriate monitoring for the occurrence of a second malignancy

## Discussion

We acknowledge the inherent limitations of analyzing retrospective studies, including the potential for publication bias. Additionally, we recognize that medical advancements, such as chimeric antigen receptor T-cell (CAR-T) therapy, are rapidly evolving and could significantly impact the landscape of treatment for hematological malignancies. Given these developments, the interpretation of kidney transplantation data, particularly in the context of previous interventions like HSCT, may not align with current or future treatment protocols.

The safety data available for many hematological malignancies listed as contraindications for kidney transplantation are very limited and, in some cases, may not be up to date. However, as novel therapies continue to demonstrate efficacy, there may be opportunities to reconsider transplant eligibility for certain malignancies in the future. While the current recommendations are based on available evidence and expert consensus, they remain a reflection of the understanding of present observations and evidence and will evolve as newer and more comprehensive data become available. As such, these recommendations are presented as a consensus report rather than formal guidelines, acknowledging that future updates may be required to incorporate emerging therapies and refine eligibility criteria.

We further acknowledge that our review is unable to provide a more granular recommendation for waitlist placement for deceased donor kidney transplantation, stratified by age or co-morbidity. Firstly, in the treatment of hematological malignancies, regimens are often adjusted to age and other co-morbidities, potentially impacting remission rates. Secondly, the adaptation of various treatment regimens for these malignancies for different age groups or those with co-morbidities may vary across centers, depending on the risk–benefit ratio assessments. Finally, there is a paucity of data on the impact of co-morbidities on outcomes post-kidney transplantation. Thus, the prognostic significance of these factors varies across different hematological malignancies, influencing cure rates to different extents. To maintain a streamlined and clinically applicable allocation framework, we prioritized sustained  remission as the primary criterion for eligibility rather than incorporating age or comorbidities, which could introduce excessive complexity into the allocation process.

Additionally, our current review focused on recommendations for waitlist placement for deceased donor kidney transplantation, and does not cover living donor kidney transplantation, where considerations of resource constraints may not apply.

Given the scarcity of deceased donor kidneys in Singapore, we acknowledge the complexity of applying and weighing competing principles of egalitarianism and utilitarianism in donor allocation, which drives our national policy to more stringent recipient criteria. These constraints are unique to each country, resulting in differences from other consensus opinions. Therefore, having considered the available evidence and factored in the distinct challenges stated above, we recognize that this consensus report is tailored to the context of Singapore’s deceased donation constraints and may not be fully generalizable to other countries or transplant allocation algorithms.

## Conclusions

Limited data exist regarding the outcomes of kidney transplantation in individuals with pre-transplant hematological malignancies. Nevertheless, we have comprehensively reviewed the evidence to establish a consensus approach considering the prolonged wait time for a deceased donor kidney. This approach aims to balance providing these patients access to transplantation while ensuring equity to the waitlisted kidney failure population to optimize the utilization of a limited resource.

Treatment paradigms for hematological diseases continue to change rapidly. As new evidence emerges after kidney transplantation, a review of currently listed and excluded hematological malignancies for future inclusion will be undertaken.

## Supplementary Information

Below is the link to the electronic supplementary material.

Supplementary file1 (DOCX 20 KB)

Supplementary file2 (DOCX 22 KB)

## Data Availability

Data sharing is not applicable to this article as this is a consensus report, and no new data were created or analyzed in this study.

## Abbreviations

AML: Acute myeloid leukemia
AL amyloidosis: Amyloid light-chain amyloidosis
ALL: Acute lymphoblastic leukemia
C3G-MG: C3 glomerulopathy with monoclonal gammopathy
CML: Chronic myeloid leukemia
CLL: Chronic lymphocytic leukemia
CT: Computed tomography
DLBCL: Diffuse large B-cell lymphoma
HL: Hodgkin lymphoma
HSCT: Hematopoietic stem cell transplantation
IPI: International Prognostic Index
LCDD: Light chain deposition disease
MM: Multiple myeloma
MGUS: Monoclonal gammopathy of undetermined significance
MGRS: Monoclonal gammopathy of renal significance
NHL: Non-Hodgkin lymphomas
OS: Overall survival
PET: Positron emission tomography
PCD: Plasma cell dyscrasias
PFS: Progression-free survival
TKI: Tyrosine kinase inhibitors
