# Supplementary material for: Deceased donor kidney transplantation in candidates with pre-transplant hematological malignancies: a literature review and recipient allocation proposal in Singapore
Source: J Nephrol. 2025 Aug 22;38(8):2041–52. doi: 10.1007/s40620-025-02381-8 (PMC12630163; doi:10.1007/s40620-025-02381-8)
Supplement: Supplementary file 1 — Supplementary file1 (DOCX 20 KB) [file 40620_2025_2381_MOESM1_ESM.docx]

**Supplemental Table 1. Reports of kidney transplants in recipients with prior acute lymphoblastic leukemia**

|  | Butcher et al.  Clin Transplant. 1999 [19] | Beitinjaneh et al.  Clin Transplant. 2010 [20] | Tsuchimoto et al.  Clin Exp Nephrol. 2019 [21] | |
| --- | --- | --- | --- | --- |
| Sex | Female | Male | Female | Female |
| Age at HSCT (years) | 27 | 17 | 23 | 13 |
| HSCT Donor | Unrelated donor | Unrelated donor | Mother | Unrelated cord blood transplantation |
| Time from HSCT to ESKD (years) | 6 | 2 | 7 | 3 |
| Etiology of ESKD | Bone marrow transplant nephropathy | Calcineurin inhibitor toxicity | Unknown | Drug-related |
| Time from ESKD to KT (years) | 1 | 4 | 1 | 3 |
| Dialysis modality pre-transplant | Peritoneal dialysis | Haemodialysis | Not mentioned | Not mentioned |
| Age at KT (years) | 34 | 23 | 31 | 19 |
| Kidney donor | Deceased donor | Deceased donor | Mother | Mother |
| Induction immunosuppression | Not stated | Not stated | None | Basiliximab |
| Maintenance immunosuppression | Tacrolimus, Prednisone,  Mycophenolate  Mycophenolate subsequently stopped | Not stated | Prednisone,  Mycophenolate.  Both were weaned off at three months | Tacrolimus, Prednisone,  Mycophenolate |
| Follow-up post KT (years) | 1.3 | Not stated (Lost to follow-up after discharge) | 9.1 | 2.3 |
| Allograft loss | No | No | No | No |
| Patient survival | Yes | Yes | Yes | Yes |
| Other Complications | Neutropenia,  CMV pneumonia with ARDS, E. coli sepsis | None | None | Acute enteritis |

Abbreviations: ARDS Acute Respiratory Distress Syndrome; CMV Cytomegalovirus; ESKD End-stage kidney disease; HSCT Haematopoietic stem-cell transplantation; KT Kidney Transplant
