# Supplementary material for: Deceased donor kidney transplantation in candidates with pre-transplant hematological malignancies: a literature review and recipient allocation proposal in Singapore
Source: J Nephrol. 2025 Aug 22;38(8):2041–52. doi: 10.1007/s40620-025-02381-8 (PMC12630163; doi:10.1007/s40620-025-02381-8)
Supplement: Supplementary file 2 — Supplementary file2 (DOCX 22 KB) [file 40620_2025_2381_MOESM2_ESM.docx]

**Supplemental Table 2. Reports of kidney transplants in recipients with prior chronic myeloid leukemia**

|  | Butcher et al.  Clin Transplant. 1999 [19] | | Beitinjaneh et al.  Clin Transplant. 2010 [20] | Martino et al.  Leuk Res Rep. 2013 [32] | Shinoda et al. Transplant. Proc. 2020 [33] | Thiem et al.  Am J Transplant. 2021 [34] |
| --- | --- | --- | --- | --- | --- | --- |
| Sex | Female | Male | Male | Female | Male | Male |
| CML treatment before KT | HSCT (sister) | HSCT (sister) | HSCT (unrelated) | Imatinib > Dasatinib > Nilotinib | Imatinib > Dasatinib | Imatinib |
| Time from CML diagnosis to ESKD (years) | 11.7 | 7.8 | 12 | 4.8 | 10.3 | 1.3 |
| Etiology of ESKD | Bone marrow transplant nephropathy | Anti-tubular basement membrane nephritis | Unknown | Acute tubular necrosis and TTP | Declined biopsy  (Possible Imatinib-related nephropathy) | Reflux nephropathy  followed by chronic TCMR* |
| Time from ESKD to KT (years) | 0.1 | 1.2 | Not mentioned | 3.4 | 2.4 | 7 |
| Dialysis modality pre-transplant | Haemodialysis | Peritoneal dialysis | Not mentioned | Hemodialysis, then peritoneal dialysis | Haemodialysis | Haemodialysis |
| Age at KT (years) | 37 | 50 | 53 | 32 | 71 | 55* |
| Kidney donor | Living donor (sister – HSCT Donor) | Living donor  (non-HSCT donor) | Deceased (cardiac + kidney transplant) | Living donor (mother) | Living donor | Deceased donor |
| Induction immunosuppression | Not stated | Not stated | ATG | Basiliximab | Basiliximab | Basiliximab |
| Maintenance immunosuppression | None | Tacrolimus, Prednisone,  Mycophenolate | Cyclosporine, Prednisone,  Mycophenolate  Cyclosporine subsequently stopped with the use of Daclizumab | Tacrolimus, Prednisone,  Mycophenolate  Prednisolone subsequently stopped | Tacrolimus, Prednisone,  Mycophenolate, Everolimus  Everolimus subsequently stopped | Tacrolimus, Prednisone,  Mycophenolate |
| Follow-up post KT (years) | 1.3 | 1.7 | 0.5 | 0.7 | 3.3 | 2 |
| Allograft loss | No | No | No | No | No | No |
| Patient survival | Yes | Yes | Yes | Yes | Yes | Yes |
| Other Complications | None | Acute rejection treated with Muromonab-CD3 (OKT3) | Mild cardiac rejection requiring ATG  CNI-related TTP | No  Remains in molecular remission on Nilotinib | Switched to Nilotinib  Remains in molecular remission | Remains on Imatinib and in deep molecular remission |

Abbreviations: ATG Anti-thymocyte globulin; CNI Calcineurin inhibitor; ESKD End-stage kidney disease; HSCT Haematopoietic stem-cell transplantation; KT Kidney Transplant; TTP Thrombotic thrombocytopenic purpura

*Retransplant. Kidney failure due to reflux nephropathy with 1st kidney transplant from 1999-2010. Developed CML in 2016. 2nd transplant in 2017
